# Expression of type I interferon-associated genes at antiretroviral therapy interruption predicts HIV virological rebound

**DOI:** 10.1038/s41598-021-04212-9

**Published:** 2022-01-10

**Authors:** P. Zacharopoulou, E. Marchi, A. Ogbe, N. Robinson, H. Brown, M. Jones, L. Parolini, M. Pace, N. Grayson, P. Kaleebu, H. Rees, S. Fidler, P. Goulder, P. Klenerman, J. Frater

**Affiliations:** 1 Peter Medawar Building for Pathogen Research, Nuffield Department of Medicine, University of Oxford, Oxford, UK; 2 Department of Paediatrics, University of Oxford, Oxford, UK; 3 Medical Research Council/Uganda Virus Research Institute, Entebbe, Uganda; 4 Wits Reproductive Health and HIV Institute of the University of the Witwatersrand in Johannesburg, Johannesburg, South Africa; 5 Division of Medicine, Wright Fleming Institute, Imperial College, London, UK; 6 Imperial College NIHR Biomedical Research Centre, London, UK; 7 National Institute of Health Research Biomedical Research Centre, Oxford, UK

**Keywords:** Gene expression, RNA sequencing, Infectious diseases, HIV infections

## Abstract

Although certain individuals with HIV infection can stop antiretroviral therapy (ART) without viral load rebound, the mechanisms under-pinning ‘post-treatment control’ remain unclear. Using RNA-Seq we explored CD4 T cell gene expression to identify evidence of a mechanism that might underpin virological rebound and lead to discovery of associated biomarkers. Fourteen female participants who received 12 months of ART starting from primary HIV infection were sampled at the time of stopping therapy. Two analysis methods (Differential Gene Expression with Gene Set Enrichment Analysis, and Weighted Gene Co-expression Network Analysis) were employed to interrogate CD4+ T cell gene expression data and study pathways enriched in post-treatment controllers versus early rebounders. Using independent analysis tools, expression of genes associated with type I interferon responses were associated with a delayed time to viral rebound following treatment interruption (TI). Expression of four genes identified by Cox-Lasso (*ISG15*, *XAF1*, *TRIM25* and *USP18*) was converted to a Risk Score, which associated with rebound (*p* < 0.01). These data link transcriptomic signatures associated with innate immunity with control following stopping ART. The results from this small sample need to be confirmed in larger trials, but could help define strategies for new therapies and identify new biomarkers for remission.

## Introduction

Most people with HIV who are receiving antiretroviral therapy (ART) will experience rebound viraemia if they stop treatment. However, a small proportion of ART-treated individuals can stop therapy and maintain undetectable viremia—or ‘post-treatment control’ (PTC)—for months and, in some cases, years^1–3^. Other individuals spontaneously suppress HIV viraemia in the absence of therapy and maintain undetectable viral loads for many years. These ‘elite controllers’ or ‘long-term non-progressors’ are likely protected by strong T cell responses restricted by protective HLA alleles^4^. Identifying biomarkers which predict outcomes following TI would provide enormous value to both understanding mechanisms of PTC and identifying new drug candidates. Multiple clinical factors and molecular biomarkers which correlate with time to rebound have been proposed to elucidate these mechanisms^1,5–10^, however the host factors that affect the responsiveness of T cells have not yet been thoroughly characterised^11^, especially at a transcriptome level.

SPARTAC (Short Pulse Antiretroviral Treatment at HIV-1 Seroconversion)^12^ was one of the largest randomized clinical trials to study different durations of ART administered to participants with Primary HIV Infection (PHI) and which included a TI. Previous analyses revealed that although the majority of participants experienced HIV viral rebound within 4 weeks of stopping ART, some maintained undetectable viraemia to the end of the study (> 500 days after TI)^6,7^. We studied participants who received 12 months of ART started in primary HIV infection (PHI), and analysed CD4+ T cell mRNA at the time of TI. Our aim was to identify genes or gene-sets expressed by CD4+ T cells that might associate with longer periods of post-treatment control.

## Results

### Clustering of participants based on clinical response

Clustering analysis of RNA expression of SPARTAC trial participants revealed distinct groups based on gender and viral subtype (Supplementary Figure S1). Therefore, to avoid potential confounding effects, we only included female participants in this analysis. We identified 14 female participants who had received 12 months of ART commenced during PHI followed by TI. All had viable samples of PBMCs taken at the point of TI (Week 48) and for 11 participants samples were also available at pre-therapy Baseline (Week 0). Viral rebound was reported if viral load post-TI was measured > 400 copies/ml in two consecutive visits. ‘Days to viral load rebound after TI’ was used to define clinical phenotypes and structure the comparative analysis (Table 1).

**Table 1:** Participant demographics.

	Week 0 (n = 11)	Week 48 (n = 14)
Sex		
Female	11	14
Male	–	–
Country		
South Africa	11	12
UK	–	1
Uganda	–	1
Rebound group		
Early rebounder (ER)—< 100 days	4	5
Post treatment controller (PTC) > 100 days	7	9

Number of participants with samples available for analysis at Week 0 and 48. Numbers of participants analysed by sex, country of origin and clinical rebound timing.

Based on observations in SPARTAC and other cohorts^3,7,13,14^ we defined early rebounders (ER) following TI (rebound < 100 days) and post-treatment controllers (PTC) (rebound > 100 days post-TI). HLA Class I typing for these participants (Supplementary Table S1) revealed both protective (eg HLA B*81:01, B*57:03) and deleterious (eg HLA B*58:02) alleles within the cohort, but no relationship with outcome, suggesting—based on this surrogate marker—that the CD8 T cell response was not driving the clinical phenotype. Interestingly, the B*35:01 allele was identified in 0/5 ER, but 3/10 PTCs—not a statistically significant difference, but consistent with findings from the VISCONTI cohort^15^.

### Differential Gene Expression and Gene Set Enrichment Analysis show a strong association of type I interferon pathways with sustained control of viremia

Differential Gene Expression (DGE) analysis was used to identify individual genes that were differentially expressed in certain clinical groups. From a total of 12,891 genes, those with a reported adjusted *p* value (padj) < 0.05 were considered as significantly differentially expressed. Two genes were found to be significantly differentially expressed between PTC and ER, namely *POMC* and *IFITM3*. *IFITM3,* an interferon stimulated gene involved in immune response, was upregulated in PTC whereas *POMC,* a gene which encodes a precursor polypeptide for a variety of peptide hormones, was downregulated. This small number of genes identified by DGE is not surprising in view of the sample size and participant heterogeneity.

**Figure 1:**
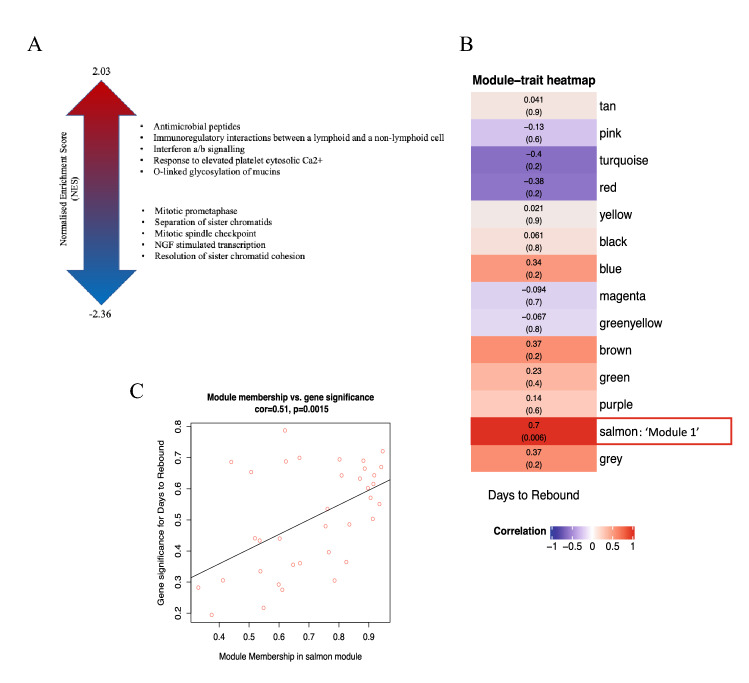
GSEA and WGCNA module identification and enrichment. (**A**) The top ten enriched pathways (FDR < 0.25) for Week 48 PTC vs ER by GSEA. Ranking is by Normalised Enrichment Score (NES). (**B**) Heatmap plot of WGCNA gene modules on TI associated with ‘days to rebound’. Trait correlations and statistical significance (in parenthesis) are shown for each module. Modules are colour-coded based on direction and intensity of correlation. The ‘salmon’ module which was identified for further analysis is marked with a box and labelled as ‘Module 1’. The heatmap was made with WGCNA (package version 1.70.3). (**C**) Scatterplot of gene significance (GS) for trait of interest versus module membership (MM) for Module 1.

To get a broader view of genetic predictors of rebound, we identified functionally linked ‘gene sets’ using Gene Set Enrichment Analysis (GSEA), which was performed on all genes ranked by their corresponding Wald statistics from the DESeq2 analysis output. Only pathways with a False Discovery Rate (FDR) < 0.25 were considered significantly enriched. The majority of pathways enriched in PTC vs ER, namely ‘Interferon alpha/beta’, ‘O-glycosylation’ and ‘response to elevated platelet pathways’, are involved with immune response regulation (Fig. 1A). Pathways involved in cell division were also found to be enriched in ER vs PTC at week 48, which might correlate with enhanced viral transcription. Of note, immune regulation pathways were not enriched at Week 0 in PTC, as they were at Week 48. However, in Week 0, there was enrichment in PTC of pathways associated with the regulation of cell-replication (Supplementary Figure S2), which may be disrupted by viral accessory proteins^16,17^, and with evidence for an association with increased production of pro-inflammatory cytokines^18^.

### Weighted Gene Co-expression Network Analysis (WGCNA) identifies module enriched in IFN-I as associated with days to rebound

For interrogation of the transcriptomics data based purely on days to rebound, Weighted Gene Co-expression Network Analysis (WGCNA) was employed to identify clusters of co-expressed genes (modules) and to inform on pathway enrichment at the point of TI in an unsupervised manner. Time to Rebound was used as a clinical trait of interest, as it offered the opportunity to explore the genetic correlations without having to make an a priori decision on sample grouping. WGCNA constructs a weighted network that represents the interaction patterns among genes, by emphasizing the strong gene–gene correlations at the expense of the weak ones in order to reduce noise. Here, the co-expression network was built using the expression data of a total of 6006 genes, that were retained after filtering for low variability and low read counts. A scale-free topology network was calculated by raising the correlation values to a power of β = 20, for which the scale-free topology fit index was above 0.8 (Supplementary Figure S3). WGCNA then clustered all genes with similar co-expression patterns into modules, conventionally denoted by colour names. A mean expression value (module eigenvalue), based on the expression of all genes within every module, was then associated with ‘Days to Rebound’ as a continuous variable.

The ‘salmon module’, named Module 1 here, had the strongest positive correlation with time to rebound (cor = 0.7, *p* = 0.006) (Fig. 1B). The module consists of 36 genes, the majority of which—for example, *IFI44, XAF1, ISG15, USP18, TRIM25, IFIT1, RSAD2*—are interferon stimulated genes (ISG). A significantly high correlation between Module Membership (MM) and Gene Trait Significance (GS) is reported for Module 1 (cor = 0.51; *p* = 0.0015), demonstrating that the genes driving the expression of the module eigenvalue, are the ones that correlate with days to rebound (Fig. 1C).

### Functional enrichment of WGCNA modules associated with PTC phenotype and Days to Rebound indicates Interferon Type I pathway involvement

**Figure 2:**
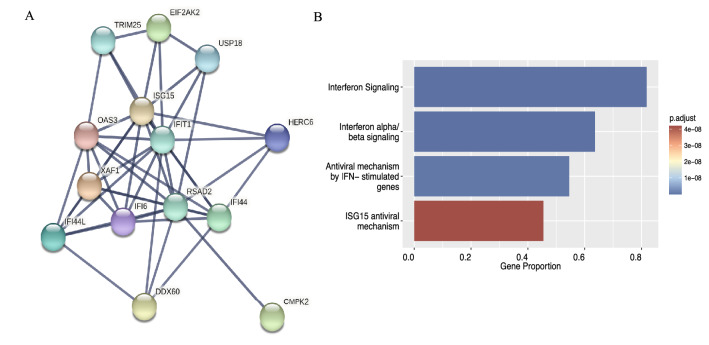
Protein Interactions and Pathways Enrichment for genes protective for viral rebound. (**A**) Visualisation of the Protein–Protein Interaction network of key genes within Module 1 using STRING for cross-validation of hub genes identified through GS and MM scoring. (**B**) Pathways enrichment bar plot identified using STRING and the Reactome database. The proportion of hub genes to pathway genes is shown on x-axis.

Protein–protein interaction (PPI) and high correlation with both module membership and days to rebound (MM and GS scores, respectively) were used as criteria to identify the hub genes, in a two-step validation process. MM and GS scores were measured by Pearson’s correlation and PPI was visualized on STRING. Figure 2A shows only the interacting module genes, after selecting for a high interaction score (> 0.9). Thirteen interacting genes, which also satisfied the GS (> 0.2) and MM criteria (> 0.2), were regarded as hub genes and were taken forward to Gene Set Enrichment Analysis. To be consistent with the GSEA, the Reactome pathway database was used to report the enrichment. Module 1 hub genes showed significant enrichment (*p* < 0.05) in pathways associated with response to IFN-I (Fig. 2B).

### Risk score calculation based on the expression of two genes can potentially predict time to rebound

As a next step, we looked to see if any of the individual hub genes identified in the WGCNA results were more closely linked to progression, potentially leading to a signature that might predict longer post-TI viral suppression. Univariable analysis showed that the expression of 7 out of 13 hub genes contained in Module 1 (*ISG15, IFI6, IFI44, RSAD2, XAF1, USP18* and *TRIM25)* were significantly associated with a protective Hazard Ratio (HR) for viral rebound (Table 2). A multivariable Cox Regression analysis was performed including these seven genes. Least Absolute Shrinkage and Selection Operator (LASSO) was used to eliminate genes with zero coefficients (*IFI6, IFI44* and *RSAD2*) and only four genes (*ISG15*; cor = -0.41, *TRIM25*; cor =—0.32, *XAF1*; cor = -0.06 and *USP18*; cor = -0.14) were retained (Supplementary Table S1).

**Table 2:** Univariable Cox regression for individual hub genes.

	HR (95% CI for HR)	*p* value
**ISG15**	**0.29 (0.11–0.81)**	**0.0091**
**IFI6**	**0.18 (0.037–0.86)**	**0.014**
IFI44L	0.55 (0.27–1.1)	0.083
**IFI44**	**0.23 (0.047–1.2)**	**0.045**
IFIT1	0.37 (0.12–1.1)	0.054
OAS3	0.52 (0.22–1.3)	0.15
**XAF1**	**0.3 (0.1–0.92)**	**0.027**
**TRIM25**	**0.074 (0.0068–0.8)**	**0.023**
CMPK2	0.23 (0.037–1.5)	0.074
**RSAD2**	**0.2 (0.041–0.99)**	**0.032**
EIF2AK2	0.54 (0.12–2.4)	0.42
**USP18**	**0.23 (0.062–0.84)**	**0.022**
HERC6	0.16 (0.019–1.3)	0.057

Hub genes identified by the WGCNA analysed contained within the ‘salmon’ Module 1. Genes in bold have *p* < 0.05 and were selected for the multivariable Cox/LASSO regression. *HR* hazard ratio, *95% CI *95% confidence intervals.

**Figure 3:**
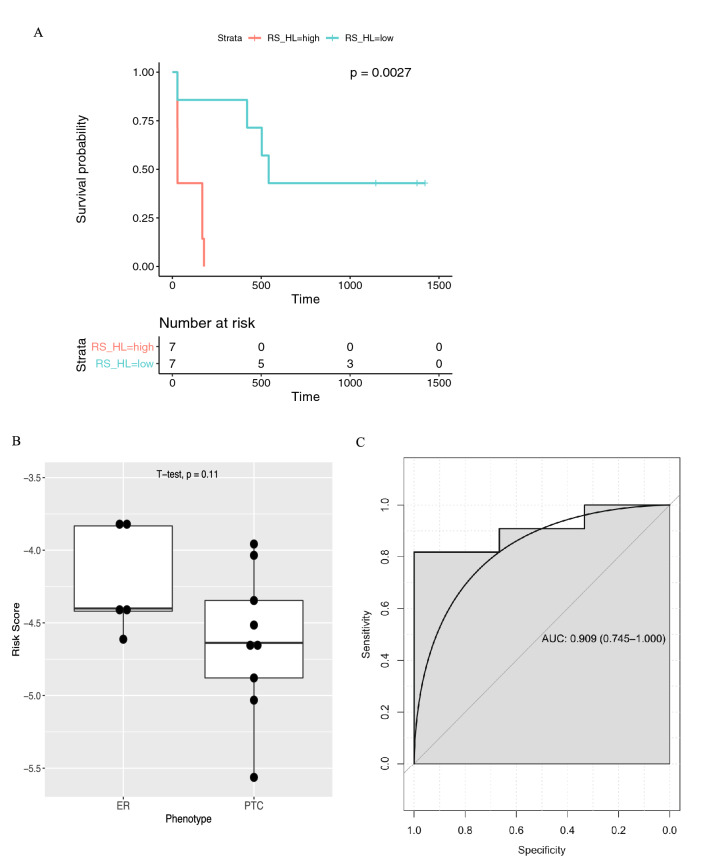
Survival analysis and gene signature validation. (**A**) Kaplan–Meier survival analysis of gene expression-based Risk Score (RS) comprising genes *ISG15, XAF1, USP18* and *TRIM25*, predicting the likelihood of early and late post-TI rebound. Blue and red lines represent low and high-risk score, respectively, divided by the mean cohort score. ‘+’ represents censored samples. (**B**) Expression of the risk score genes for each participant plotted for different phenotypes. *ER* early rebounder, *PTC* post-treatment controller. (**C**) ROC curve demonstrating efficiency of applying the Risk Score to identify participants that reported rebound versus those that did not. *AUC* area under the curve; confidence interval in brackets.

A Risk Score (RS) was calculated for the fourteen female participants using the expression of these four genes, and their LASSO coefficients were classified as either low or high relative to the mean RS (Supplementary Table S3). Kaplan Meier analysis showed that a lower RS with higher expression of *ISG15, USP18, XAF1* and *TRIM25* was significantly associated with longer suppression post-TI, compared to participants with a high RS (*p* < 0.01) (Fig. 3A). Boxplots showing the risk score for each participant in the PTC and ER groups based on expression of the Cox-LASSO derived genes (Fig. 3B) and the ROC curve (Fig. 3C) shows the effectiveness of the signature (AUC = 0.909) in distinguishing rebounders from non-rebounders.

## Discussion

The aim of the study was to gain further understanding of how host gene expression might affect the duration of post-TI viral control. The SPARTAC trial offered a unique opportunity to study samples from participants with PHI that had been followed-up for an average of four years post-TI^12^. To minimise the noise caused by averaging the expression of different cell types, we studied CD4+ T cells as they are key to virological control^19^ and comprise the majority of the HIV reservoir. Therefore, any variability in transcriptional activity might impact the likelihood of proviral expression. In addition, we restricted our analyses to women to exclude potential confounding factors from sex.

Due to the small sample size and the major variance between the phenotypes, DGE analysis identified only two genes differentially expressed between PTC and ER. IFITM3, which was upregulated in PTC, plays a critical role in actively preventing infection by inhibiting viral-cell fusion^20^. In turn, *POMC*, a precursor of melanocortins ACTH and MSH, was downregulated in PTC. Evidence to associate melanocortins with anti-inflammatory action has been reported in vivo and in vitro^21^. Downregulation of POMC in PTC may denote inflammation suppression, which may be protective against viral rebound at the early stage of TI.

The results of GSEA—which is less impacted by sample size—revealed that the PTC phenotype (i.e. rebound > 100 days after TI) showed enrichment for the Type I Interferon (IFN-I) signaling pathway as well as O-glycosylation and platelet activation pathways. Response to IFN-I was also found to be associated with days to rebound in the WGCNA approach, which made no prior assumptions about definitions of post-treatment control. IFN-I pathways comprise a family of cytokines playing a critical role in the regulation of the innate immune response in infection^22^. Studies in SIV models illustrate the benefit of IFN-I administration at the early stage of infection in inhibiting viral replication^23–25^. Similarly, HIV develops strategies to evade IFN-I and to inhibit the functionality of the proteins regulated by IFN-I^26^, suggestive of a fundamental role of this pathway in viraemic control. Previous studies have highlighted the importance of IFN-I in controlling HIV infection, by showing that founder viruses that are able to establish infection are usually IFN-resistant but may be less fit^27–29^. Recent findings from Gondim et al.^30^, showed that viral resistance to IFN-I fluctuates throughout infection and, notably, post-TI plasma viruses are the most IFN-I resistant. This suggests that the induction of IFN-I immediately after infection or treatment interruption can clear IFN-I sensitive viruses and only allow IFN-I resistant viruses to replicate. Although there is no reported association between T-cell immune escape and changes in IFN-I sensitivity, CTL escape^31,32^ has been well-described during primary infection and the possibility of this affecting IFN-I sensitivity should be investigated. O-linked glycosylation may also contribute to the regulation of the immune system and T-cell development^33^, with recent evidence of a link between type I interferons and modulation of the host glycome in HIV infection^34^. The association of the PTC phenotype with platelet activation is intriguing and possibly consistent with reports that release of chemokines by activated platelets can function as an extra barrier at the early stages of HIV infection^35^. However, further investigation is required to determine the role of platelets in the defence against HIV.

WGCNA was performed to determine how gene modules associate with specific clinical traits. For an unbiased analysis, ‘time to rebound’ was used as a trait to examine whether WGCNA findings corresponded with the GSEA analysis. The analysis identified one dominant module that was associated with ‘Time to Rebound’. The gene ontology and pathway analysis for this Module showed a clear enrichment for IFN-I associated genes, supporting the previous results for the phenotype traits. The majority of genes reported as hub genes were ISG, again consistent with the argument that the response to type I interferons is impacting rebound. None of the hub genes in the Module were found among the DE genes. This is not surprising, given that WGCNA associates gene co-expression modules with the trait of interest, rather than individual genes. Besides, DGE analysis was based on arbitrary clustering of participant phenotypes, which may have made singling out genes less robust and accurate. To this end, only genes identified with WGCNA were used to build a gene signature in the subsequent steps.

A univariable Cox analysis identified seven genes, mostly ISGs, associated with remission in the participants. Based on a multivariable Cox regression with LASSO screening of these seven genes, a risk score based on the expression of four (*ISG15, XAF1, TRIM25* and *USP18*) was derived to predict the likelihood of viral rebound. Higher expression of these four genes was strongly protective for post-TI rebound. *XAF1* is a proapoptotic tumor suppressor protein, which is induced by IFN-I and tumor necrosis factor alpha (TNFa)^36^. *ISG15* is an interferon stimulated ubiquitin-like protein and a critical component of protein modification and cell cycle regulation^37,38^. *TRIM25* is an E3 ligase that positively regulates *ISG15* conjugation to pathogen proteins, in a process with reported antiviral effects called ISGylation^39^. *USP18* is a negative regulator of IFN-I signalling that dampens its detrimental effects^38,40,41^. Although more work is underway to characterise the role of these pathways in HIV infection, this independent identification of these genes would be consistent with a role in maintaining virological remission.

The small sample size on which this analysis was performed is a key limitation. In addition, our analysis was limited to bulk CD4+ T cells, with no enrichment for those cells that were HIV-specific or contained proviral DNA. A further limitation is that due to a lack of remaining samples we have been unable to carry out any subsequent confirmatory experiments to test the significance of the genes identified, although this work is now planned for a subsequent study incorporating a TI component (The RIO Trial; ClinicalTrials.gov Identifier: NCT04319367). The other factor to consider is whether our choice of clinical phenotypes accurately discriminated between ‘post-treatment controllers’ and ‘elite controllers’. The latter have been well characterized^1^, and more generally associated with effective HLA Class I-restricted T cell responses^42^, whereas it is still unclear as to which mechanisms are driving PTC. Our analysis of HLA types does reveal a number of protective alleles, but not enough to explain our data. Larger studies will be needed to tease out these differences. However, that two independent analyses conferred the same statistically significant results of pathway enrichment should be taken into consideration. The finding of a strong type I interferon signal associating with delayed rebound in this small study needs to be confirmed in larger clinical trials incorporating TI. If these data are reproducible, they could help with the identification of a valuable biomarkers of remission and pathways to drug discovery for the HIV cure field.

## Methods

### Participant characteristics and study design

SPARTAC (EudraCT Number: 2004-000446-20) was a multi-centre randomised controlled trial of short course antiretroviral therapy during PHI, which completed follow up in 2010. The full inclusion criteria and details of the SPARTAC trial are published elsewhere^12^. All participants gave full informed consent. We initially analysed 18 SPARTAC participants who had received 48 weeks of suppressive ART commenced during Primary HIV Infection (PHI). Participants were selected based on availability of viable peripheral blood mononuclear cells (PBMCs) and stratified by time to rebound (> 400 HIV RNA copies/ml of plasma). Participants were sampled at Week 0 ‘Baseline’ at the time of starting ART and at the time of TI, 48 weeks later. The demographics of the participants are presented in Table 1, and Supplementary Table S1. Following a pre-analysis step, fourteen female participants were selected for further analysis, to decrease the impact of confounding variables, specifically the linear relationship between sex and viral clade as well as the lack of PTC phenotypes in male participants.

### Ethics statement

The SPARTAC trial, and the experimental analyses performed in this study were approved by the following authorities: the Medicines and Healthcare products Regulatory Agency (UK), the Ministry of Health (Brazil), the Irish Medicines Board (Ireland), the Medicines Control Council (South Africa) and the Uganda National Council for Science and Technology (Uganda). It was also approved by the following ethics committees in the participating countries: the Central London Research Ethics Committee (UK), Hospital Universitário Clementino Fraga Filho Ethics in Research Committee (Brazil), the Clinical Research and Ethics Committee of Hospital Clinic in the province of Barcelona (Spain), the Adelaide and Meath Hospital Research Ethics Committee (Ireland), the University of Witwatersrand Human Research Ethics Committee, the University of Kwazulu-Natal Research Ethics Committee and the University of Cape Town Research Ethics Committee (South Africa), Uganda Virus Research Institute Science and ethics committee (Uganda), the Prince Charles Hospital Human Research Ethics Committee and St Vincent’s Hospital Human Research Ethics Committee (Australia) and the National Institute for Infectious Diseases Lazzaro Spallanzani, Institute Hospital and the Medical Research Ethics Committee, and the ethical committee of the Central Foundation of San Raffaele, MonteTabor (Italy). All experiments were performed in accordance with relevant guidelines and regulations and all participants signed a written informed consent.

### Analytical approach

Taking into consideration the limitations of the sample size, two approaches were taken. Firstly, a Differential Gene Expression (DGE) followed by Gene Set Enrichment Analysis (GSEA) was carried out, aiming to identify signalling pathways enriched in pre-defined clinical phenotypes. Next, a Weighted Gene Co-expression Network Analysis (WGCNA) was performed to detect pre-defined gene “modules” without a priori phenotype characterization and investigate their association with days to rebound. A flow diagram of this analysis is shown in Supplementary Figure S4. For DGE, participants who rebounded > 100 days after TI were classified as post-treatment controllers (PTC) and those who rebounded earlier than 100 days were classified as early rebounders (Table 1). Initial analyses revealed a co-linear relationship between sex and ethnicity/viral subtype (all but one men were HIV subtype B, and all but one women were subtype C) and so—to avoid confounders—this study was restricted to the 14 female participants.

### RNA isolation and sequencing

CD4+ T cells were isolated using negative selection (Stem Cell Technologies CD4 enrichment kit) according to the manufacturer’s recommendations. Total RNA was extracted using the Qiagen RNA Kit with Qiashredder columns. Material was quantified using RiboGreen (Invitrogen) on the FLUOstar OPTIMA plate reader (BMG Labtech) and the size and integrity analysed on the 2200 TapeStation (Agilent, RNA ScreenTape). Input material was normalised to 100 ng prior to library preparation. Polyadenylated transcript enrichment and strand specific library preparation was completed using NEBNext Ultra II mRNA kit (NEB) following manufacturer’s instructions. Libraries were amplified on a Tetrad (Bio-Rad) using in-house unique dual indexing primers^43^. Individual libraries were normalised using Qubit, and the size profile analysed on the 2200 TapeStation. Sequencing was performed using an Illumina Novaseq6000 platform at 150 paired end mode (Illumina, San Diego, CA).

### Differential gene expression screening and Gene Set Enrichment Analysis

DESeq2^44^ was used to compute differential gene expression (DGE) between phenotypes using a featurecounts table. The dataset was filtered for low count reads (< 10 reads in 60% of samples). Only genes with a DGE of adjusted *p* value of < 0.05, after adjusting for multiple testing using Benjamini-Hochberg, and |log_2_ fold change|> 1 were considered statistically significant. Gene Set Enrichment Analysis (GSEA)^45,46^ was used to detect differences in biologically relevant pathways in the dataset. The datasets were pre-ranked by the DESeq2 Wald statistic value. Gene set permutation was set at 1000. Gene sets in the peer-reviewed Reactome pathway database^47^ were used as reference. Results satisfying a False Discovery Rate (FDR) cut off of < 25% were considered statistically significant.

### Co-expression network construction

Weighted Gene Co-expression Network Analysis (WGCNA)^48,49^ is an R package used for gene expression profiling and was applied to the identification of genes associated with time to rebound. After pre-processing for low variance filtering and outlier removal, an appropriate soft-threshold power was selected to promote and penalise the strong and weak gene connections, respectively. Following this, a signed network was created using the one-step approach, according to the package manual. Genes were organised in modules, based on a common expression pattern and a colour-label was attributed to each to assist identification. The expression of the genes in each module was summarised in an eigenvalue, which was then correlated with the trait of interest to identify the most biologically relevant modules.

### Identification and annotation of important modules and Hub genes

The modules that were selected for downstream analysis were the ones that had a significant correlation with the trait. The most connected genes (hub genes), which are of functional significance, were defined by their Module Membership (MM > 0.8) and their Gene-trait Significance (GS > 0.2) measured with Pearson correlation. The module genes were also uploaded on STRING^50^, an online tool for pathway enrichment, gene ontology annotation and PPI visualization in order to cross-validate the hub genes. Only genes that satisfied the connectivity as well as GS and MM score criteria were considered true hub genes.

### Identification of predictor genes for time to rebound

Survival analysis was performed for the identified hub genes using R^51^. The first viral rebound after TI was used as the event of interest. A univariable Cox Proportional Hazard Regression was performed on each gene. Genes with a statistically significant correlation to time to rebound were selected for a multivariable Cox regression with LASSO after selecting an optimal regularization parameter λ (× 1000 repetitions), to shrink the variable coefficients. A Risk Score (RS) to predict prognosis was calculated according to the formula below, where β is the multivariate Cox coefficient and exp is the expression value for all significant genes.

$$RS = \mathop \sum \limits_{n = 1}^{gene} \beta_{gene} \times exp_{gene}$$

All participants that did not report post-TI rebound during the follow-up period of SPARTAC were classified as censored. All genes with a statistically significant association with a longer remission were then used to calculate a prediction score for time to rebound, by multiplying the gene coefficient with the gene expression.

## Supplementary Information

Supplementary Information.
