# Supplementary material for: Expression of type I interferon-associated genes at antiretroviral therapy interruption predicts HIV virological rebound
Source: Sci Rep. 2022 Jan 10;12:462. doi: 10.1038/s41598-021-04212-9 (PMC8748440; doi:10.1038/s41598-021-04212-9)

Expression of type I interferon-associated genes at antiretroviral therapy interruption predicts HIV virological rebound

P. Zacharapoulou[1*], E. Marchi[1*], A. Ogbe[1], N. Robinson[1], H. Brown[1], M. Jones[1], L. Parolini[1], M. Pace[1], N. Grayson[1,2], P. Kaleebu[3], H. Rees[4], S. Fidler[5,6], P. Goulder[2], P. Klenerman[1,7], J. Frater[1,7†]

[1] Peter Medawar Building for Pathogen Research, Nuffield Department of Medicine, University of Oxford, Oxford, UK

[2] Department of Paediatrics, University of Oxford, Oxford, UK

[3] Medical Research Council/Uganda Virus Research Institute, Entebbe, Uganda

[4] Wits Reproductive Health and HIV Institute of the University of the Witwatersrand in Johannesburg, South Africa

[5]Division of Medicine, Wright Fleming Institute, Imperial College, London, UK

[6] Imperial College NIHR Biomedical Research Centre, London, UK

[7] National Institute of Health Research Biomedical Research Centre, Oxford, UK

[†]Correspondence to: John Frater; Peter Medawar Building for Pathogen Research, Nuffield Department of Medicine, University of Oxford, Oxford, UK. john.frater@ndm.ox.ac.uk

*These authors contributed equally

Supplementary Materials

Supplementary Table 1: Detailed Demographics

Study ID	Sex	Age	Category	Days to Rebound	Country	Viral Clade	HLA B Types	Pre-ART Viral Load (log)
SIU042002	Male	35	ER	24	Italy	D	-	5.69
SJR026007	Female	31	ER	28	South Africa	C	B*41:01, B*42:01	5.29
SJZ026003	Female	40	ER	28	South Africa	C	B*15:03, B*42:01	4.78
SUN036016	Female	23	ER	28	UK	C	B*42:02, B*51:01	3.68
SUU036030	Male	46	ER	28	UK	A/B	B*44:02, B*44:02	4.82
SJA023044	Female	48	ER	29	South Africa	C	B*15:10, B*58:02	4.06
SJS025009	Female	23	ER	29	South Africa	C	-	4.78
SJK023006	Female	20	PTC	168	South Africa	C	B*44:02, B*81:01	4.49
SJV101017	Female	49	PTC	168	Uganda	C	-	2.83
SJJ027008	Female	25	PTC	178	South Africa	C	B*8:01, B*13:02	3.11
SUA038008	Male	34	PTC	178	UK	AG/B	B*8:01, B*35:01	3.11
SUX010001	Male	27	PTC	301	UK	B	B*45:01, B*51:01	5.01
SJH027017	Female	22	PTC	420	South Africa	C	B*35:01, B*57:03	2.60
SJL026018	Female	24	PTC	503	South Africa	C	B*44:03, B*58:02	5.64
SJC030002	Female	28	PTC	542	South Africa	C	B*15:10, B*58:02	4.82
SJE021010	Female	21	PTC	1145	South Africa	C	B*7:02, B*39:10	4.26
SJE030001	Female	27	PTC	1376	South Africa	C	B*8:01, B*15:03	2.66
SJV026005	Female	21	PTC	1421	South Africa	C	B*35:01, B*81:01	2.60

Supplementary Table 2: Gene expression coefficients for genes detected with Cox/LASSO.

**Gene Name**	**Coefficient**
ISG15	-0.41
IFI6	0
TRIM25	-0.32
IFI44	0
XAF1	-0.06
RSAD2	0
USP18	-0.14

Genes with non-zero coefficients were selected to construct a Risk Score to predict time to post-TI rebound.

Supplementary Table 3: Individual Risk Score per participant, based on the expression of gene signature

Participant	Days to Rebound	Incidence	Risk Score (RS)
SJV026005	1421	0	-5.56
SJC030002	542	1	-5.01
SJE030001	1376	0	-4.88
SJH027017	420	1	-4.67
SJE021010	1145	0	-4.64
SUN036016	28	1	-4.61
SJL026018	503	1	-4.51
SJS025009	29	1	-4.42
SJZ026003	28	1	-4.40
SJV101017	168	1	-4.34
SJJ027008	178	1	-4.03
SJK023006	168	1	-3.96
SJR026007	28	1	-3.83
SJA023044	29	1	-3.81
	Average Risk Score		-4.48

Supplementary Figure 1. Participant clustering based on gene expression

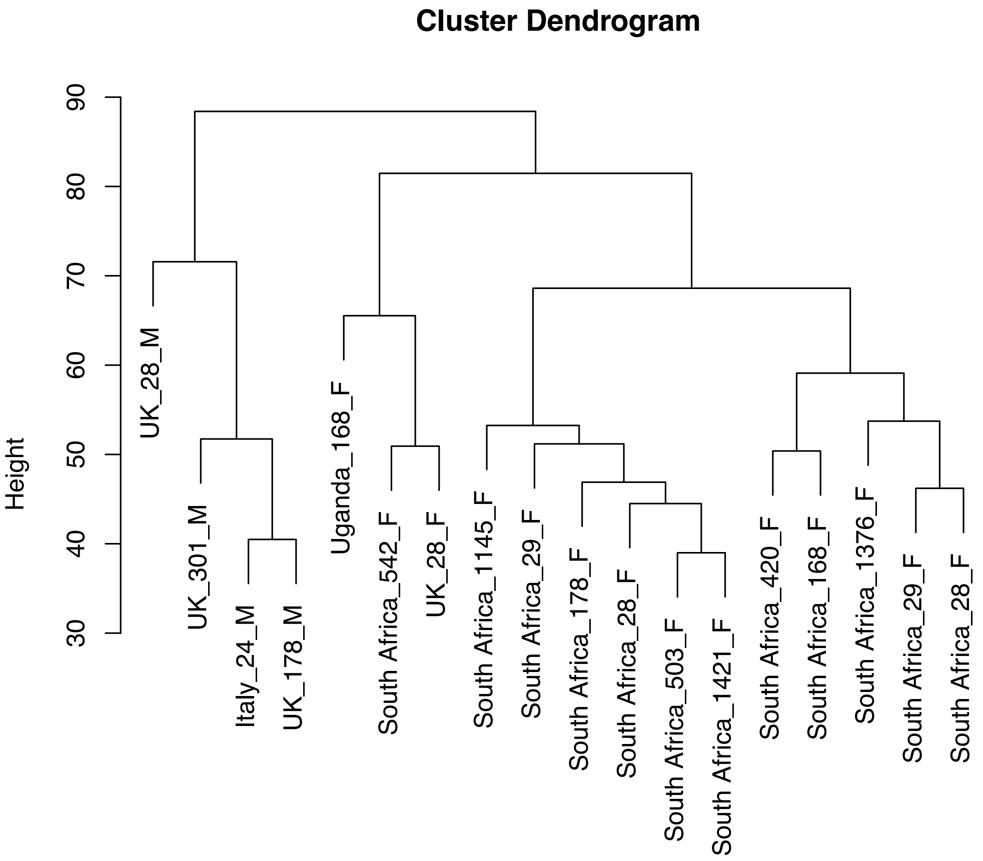

Supplementary Figure 2. Week 0 pathway enrichment in PTC vs ER according to clinical phenotype using GSEA and the Reactome pathway database.

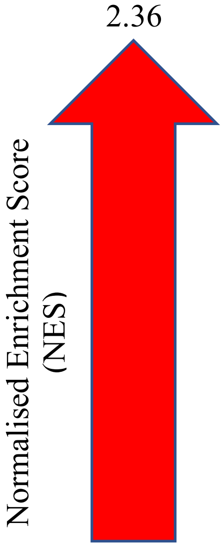

- Activation of ATP in response to replication stress
- Activation of the pre-replication complex
- Polo-like kinase-mediated events
- G1-S specific transcription
- Cell cycle checkpoints

Supplementary Figure 3. Network topology analysis for different soft thresholding powers and mean connectivity.

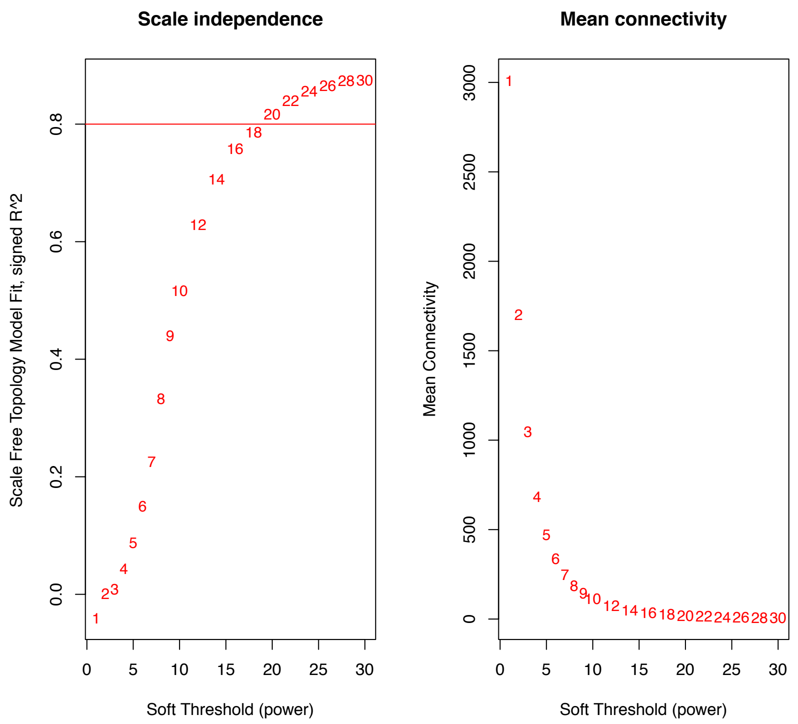

A power of 20 was selected to achieve scale-free topology of $R^2 > 0.8$ and Mean Connectivity close to 0.

Supplementary Figure 4: Flow diagram of the study analysis

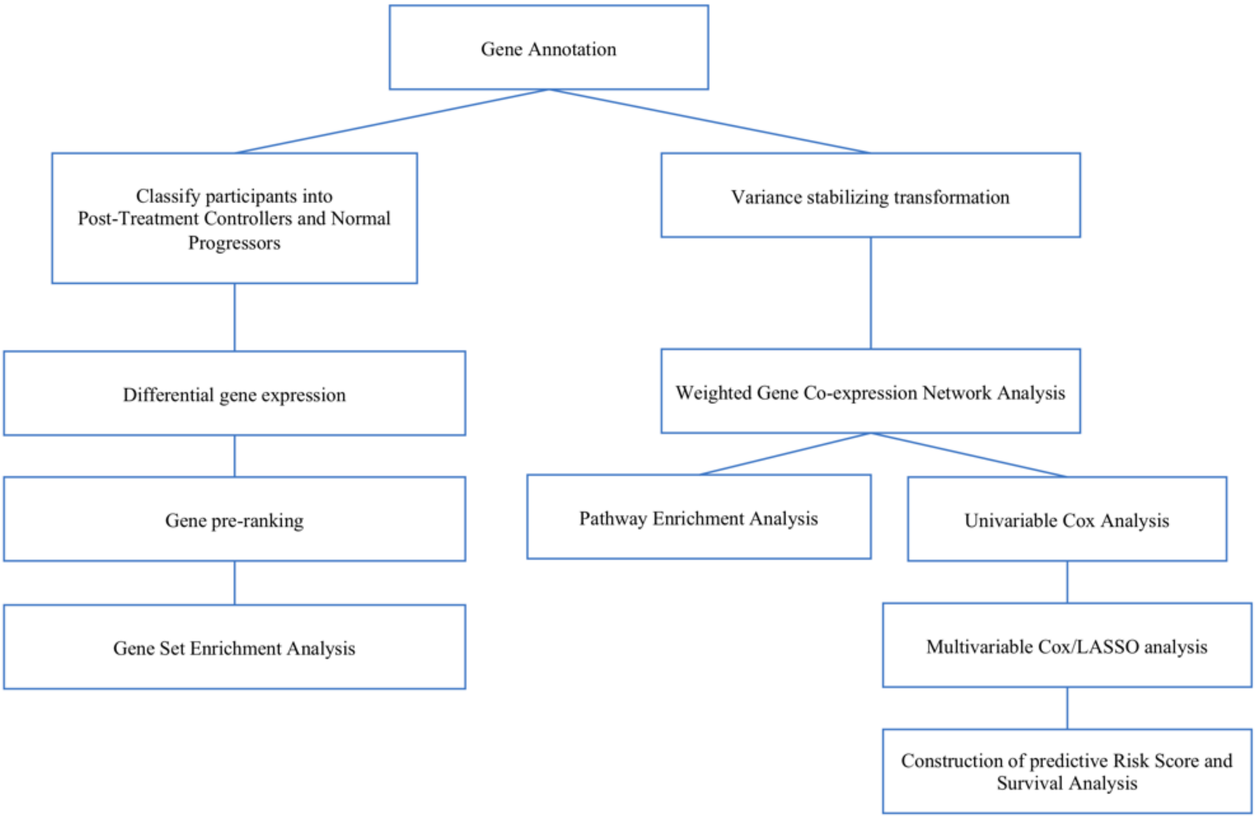

Supplement: Supplementary file 1 — Supplementary Information. [file 41598_2021_4212_MOESM1_ESM.pdf]
